# Scenes in the Practice of a New York Surgeon

**Published:** 1855-11

**Authors:** 


*Scenes in the Practice of a New York Surgeon.* By Edward H. Dixon, M. D., Editor of the Scalpel.

This book was sent us for notice, and we received it with a premonitory feeling that we should be compelled to notice it unfavorably. We did not like the source from whence it came. We have always looked upon Dr. Dixon as a man of talent and professional attainment, but one grievously gone astray in his ideas of what was due his profession and its character. The abuse he has heaped upon the physicians of New York was too lavish to be truthful — we could not believe that his harsh opinions were fully justified. Admitting that they were, we did not recognize the propriety of the manner of the attack.

With such preconceived notions it was with no favoreabl impression that we opened the pages of this book. A perusal, however, leads us to other ideas. Aside from occasional ebullitions of feeling against his old enemies, (and these are deprived of all personality) he has given us a volume containing much of that sad and truthful pathos which marks the physician's life; much graphic description of different phases of human nature; much indignant remonstrance against the shams of American society, and much advice to general readers, of a good and wholesome character.

We hope it may have a general circulation. It is calculated to do us—the profession—good with the people, and to convey correct ideas of our position and duties. Aside from all this it has the merit—indispensable to a book for the general reader—of a fine, easy, and graphic style, which charms and interests the mind. It is for sale by BURKE—under the Mansion House.

